# Key outcomes in treatment of activated phosphoinositide 3-kinase delta syndrome: An e-Delphi panel study and responder threshold application

**DOI:** 10.1371/journal.pone.0333341

**Published:** 2025-10-15

**Authors:** Julia E. M. Upton, Kelli W. Williams, Andrew Cant, Ana Santos, João Bana e Costa, Jason Bradt, Amanda Harrington, Chad Gwaltney

**Affiliations:** 1 Division of Immunology and Allergy, Department of Paediatrics, The Hospital for Sick Children, Toronto, Ontario, Canada; 2 Department of Paediatrics, Temerty School of Medicine, University of Toronto, Toronto, Ontario, Canada; 3 Medical University of South Carolina, Charleston, South Carolina, United States of America; 4 Newcastle University, Newcastle upon Tyne, United Kingdom; 5 CEGIST, Instituto Superior Técnico, Universidade de Lisboa, Lisbon, Portugal; 6 Decision Eyes Lda, Lisbon, Portugal; 7 Pharming Healthcare, Inc., Warren, New Jersey, United States of America; 8 Gwaltney Consulting, Westerly, Rhode Island, United States of America; Laval University, CANADA

## Abstract

**Background:**

Activated phosphoinositide 3-kinase delta syndrome (APDS) is an ultra-rare, underrecognized inborn error of immunity. This study aimed to identify outcomes important in evaluating APDS treatment effectiveness and percent change in specific outcomes indicating a clinically meaningful benefit.

**Methods:**

In this e-Delphi panel study, 28 globally based APDS experts used a 5-point Likert scale (Strongly Disagree to Strongly Agree) to indicate level of agreement that an outcome was an important measure of APDS treatment effectiveness in adult and pediatric patients at 3 and 6 months after treatment initiation. A threshold of ≥75% responding with “Agree” or “Strongly Agree” was considered consensus. Percent meaningful improvement in 6 outcomes was assessed and applied to APDS trial data (NCT02435173).

**Results:**

Twenty-four panelists participated; e-Delphi rounds 1–5 were completed by 23, 21, 18, 17, and 16 panelists, respectively. Outcomes with the highest degree of consensus included lymph node size/volume, clinician overall impression of disease activity, antibiotic use, patient/caregiver-reported social outcomes and patient quality of life, hospitalizations, thrombocytopenia, spleen volume, lymphopenia, and anemia. Panelists indicated within-patient clinically meaningful improvements in adult patients ranged from median values of 20%−25% in lymph nodes, naïve B-cell to total B-cell ratio, spleen volume, hemoglobin, platelets, and lymphocytes at 3 months, and 25%−30% at 6 months. Panelists indicated within-patient clinically meaningful improvements in pediatric patients ranged from median values of 20%−27.5% at 3 months and 22.5%−45% at 6 months in the same 6 outcomes. In an application of responder thresholds, treatment with leniolisib resulted in significant and meaningful improvements in disease hallmarks, including lymph node size, spleen volume, and naïve B-cell ratio.

**Conclusion:**

This study provides expert consensus on outcomes important in assessing APDS treatment effectiveness and improvement thresholds in 6 treatment outcomes indicative of a clinically meaningful benefit. These outcomes may help optimize APDS treatment in the clinic.

## Introduction

Activated phosphoinositide 3-kinase delta (PI3Kδ) syndrome (APDS) is an ultra-rare, underrecognized inborn error of immunity (IEI) that was first characterized in 2013 [1,2]. As of March 2023, a total of 351 unique patients with APDS have been reported globally in the literature [3]. The rarity of this disease is further underscored by the fact that the estimated global population reached 8.01 billion in 2023 [4]. Despite improved awareness and genetic diagnostics, the APDS prevalence reported in the literature remains far less than the < 1 per 50,000 people that defines an ultra-rare disease [1–3]. As an IEI, APDS results from genetic variants [5,6]. Pathogenic, heterozygous variants occur in 1 of 2 genes encoding the PI3Kδ heterodimer: gain-of-function variants in phosphatidylinositol-4,5-bisphosphate 3-kinase catalytic subunit delta (*PIK3CD*) encoding the catalytic subunit p110δ cause APDS1, and loss-of-function variants in phosphoinositide-3-kinase regulatory subunit 1 (*PIK3R1*) encoding the regulatory subunit p85α cause APDS2 [7–12]. These pathogenic variants lead to hyperactivation of PI3Kδ signaling in immune cells and result in immune deficiency and immune dysregulation in a wide range of immune cells [7,13,14].

APDS-related manifestations often occur early in life, with a diverse set of clinical phenotypes and symptoms as well as severity that may vary considerably between patients [1]. Immune dysregulation with APDS, including abnormal antibody production, may lead to manifestations, including recurrent respiratory infections, herpesvirus infections, nonmalignant lymphoproliferation (e.g., lymphadenopathy, splenomegaly, and hepatomegaly), autoimmunity, and enteropathy [7,14–17]. Increased risk of malignancy has also been reported in patients with APDS, including lymphoma in up to 28% of patients and other malignancies (e.g., leukemia and dysgerminoma) in 2.8% of patients [3,15]. Long-term outcomes associated with APDS may include multiorgan damage or failure, and emerging evidence is suggestive of neurodevelopmental delays [18]. An overall survival analysis estimated that patients with APDS experience early mortality relative to the global population; the median age of survival was decreased by 11 years in patients with APDS [19]. This separation in overall survival began in adolescence and continued throughout adulthood [19]. These outcomes highlight the need for early diagnosis and implementation of effective therapies that address the underlying cause of APDS to potentially change the trajectory of this disease.

To our knowledge, there are no guidelines defining recommendations for APDS treatment outcomes. Thus, defining treatment effectiveness in APDS is challenging. Due to heterogeneity in clinical presentation and diversity of biomarkers, it is key to identify variables that reflect the most important treatment outcomes for patients as well as to characterize clinically meaningful responses in individual patients to guide treatment.

This study was designed to identify the most important treatment outcomes in APDS and to estimate the thresholds for meaningful within-patient change through an online e-Delphi panel study. This information will help guide clinical research in APDS and related areas, including other IEI.

## Materials and methods

The study included 2 phases with expert clinicians: clinician interviews and an e-Delphi panel study. Clinician interviews were conducted before initiation of the e-Delphi panel study and provided initial information on treatment outcomes that was incorporated into the online surveys that were administered in the e-Delphi panel study.

### Clinician interviews

In 2022, 9 clinicians from the United States and Europe were invited to participate in the interviews. The goal was to solicit responses from individuals with specific expertise in APDS, lymphadenopathy, and B-cell disorders. Clinicians in the latter 2 categories could have specialties from areas outside of immunology. Nine clinicians accepted the invitation and participated in the interviews. Interviews were conducted via 45- to 60-minute videoconferencing sessions using a semi-structured interview guide. Segments of the interview guide addressed the following: (1) important APDS treatment outcomes 3 and 6 months after treatment initiation, (2) meaningful change thresholds on the log_10_-transformed sum of the perpendicular diameters (SPD) of index lymphoid tissues (i.e., lesions) and ratio of naïve B cells to total B cells metrics, (3) a multidomain response index (MDRI) that incorporated multiple outcomes (e.g., SPD of index lymph nodes, percentage of naïve B cells/total B cells, transitional B cells, spleen volume, erythrocyte sedimentation rate [ESR], and patient global impression) into a patient profile, and (4) a comprehensive listing of outcomes and biomarkers that were collected in an APDS phase 3 trial [20]. Across these segments, clinicians were asked to identify outcomes that are most important to evaluate the effectiveness of an APDS treatment in pediatric and adult patients at 3 and 6 months after start of treatment. Notes collected from each interview were assessed by thematic analysis, in which each segment of the interview notes was evaluated and coded. The frequency of each code was generated, and general themes were extracted.

As consistent thresholds were not identified during the clinician interviews, it was determined that a Delphi panel study was needed to obtain consensus. Themes from the clinician interviews were used to develop the initial survey presented in the e-Delphi panel study.

### E-Delphi panel study

Clinicians were eligible to participate on the e-Delphi panel if they (1) were currently treating ≥1 patient with APDS, (2) had previously treated ≥1 patient with APDS, or (3) were currently treating ≥1 patient with a primary immunodeficiency (PI). Twenty-eight clinicians from the United States, Canada, the United Kingdom, and Italy were invited to participate in the e-Delphi panel study.

Following completion of the clinician interviews, evidence from available clinical trial protocols was reviewed and clinical insight was sought regarding treatment outcomes before beginning design of the e-Delphi panel study. A 5-round e-Delphi process was conducted virtually between March and November 2023, using the RAND/UCLA Appropriateness Method—an internationally recognized technique for achieving expert consensus on health practices or policy based on the opinions of a panel of experts [21,22]. The study survey rounds were administered online via the Welphi platform (https://www.welphi.com; Decision Eyes, 2023). The surveys were designed to ascertain expert opinions on outcomes in 4 subgroups: adult patients at 3 and 6 months after start of treatment and pediatric patients at 3 and 6 months after start of treatment. The online surveys applied dichotomous “yes/no” scales, a 5-point Likert rating scale (Strongly Disagree, Disagree, Neither Agree nor Disagree, Agree, or Strongly Agree), and open-ended free-text responses in which participants could provide comments about any outcome. At the completion of each survey, anonymized summaries of the results were aggregated and presented to the panelists in the subsequent round in concordance with e-Delphi recommendations [23]. In Delphi panels, controlled feedback is important, and panelists were given the option to change their original responses if their perspective had changed after seeing how other panelists had answered the same questions [23]. This anonymized system allowed each panelist to provide their opinion without undue influence from other panelists. In Rounds 3 and 4, panelists could review anonymized comments from the previous round.

#### Round 1.

In Round 1, all panelists completed introductory questions regarding their experience with APDS and PI. Next, panelists were asked to consider a listing of treatment outcomes for each patient subgroup. For example, panelists were asked the following question: “Imagine that you are evaluating how well a new treatment is working for an ADULT patient with APDS, in your practice, 3 MONTHS after starting the treatment.” Panelists could then propose the removal of ≥1 of the treatment outcome variables from the listing or enter a new outcome variable that was not included in the listing.

#### Rounds 2 and 3.

In Round 2, the results of Round 1 were summarized. Panelists were asked to indicate their level of agreement (using the 5-point Likert scale) that each treatment outcome was important for each patient subgroup. In Round 3, the Round 2 results were summarized, and panelists were asked to indicate their level of agreement. Panelists were given the option to change their original responses if their perspective had changed after seeing how other panelists had answered the same questions. Additionally, panelists were asked to indicate their level of agreement on new outcomes, which were more specific variations of the general categories that had been presented in the Round 2 survey. The same 5-point Likert scale to indicate agreement was used.

#### Round 4.

In Round 4, panelists were presented with the results of the Round 3 survey for the new outcomes and asked to indicate their level of agreement using the 5-point Likert scale. Panelists were given the option to change their original responses if their perspective had changed after seeing how other panelists had answered the same questions. Outcomes reaching consensus in Round 3 were considered finalized. In Round 4, panelists were also asked to consider selected outcome variables and indicate the percent improvement threshold that would be clinically meaningful for each patient subgroup. Outcome variables included lymph node size, naïve B-cell count, spleen volume, hemoglobin levels, platelet count, and lymphocyte count.

#### Round 5.

In Round 5, panelists were presented with an anonymized summary of the Round 4 responses and were given the opportunity to change their response for each outcome in Round 4.

#### Data analysis and definition of consensus.

The distribution of Likert scale responses was calculated for each outcome, with consensus predefined as ≥75% of panelists responding “Agree” or “Strongly Agree” [24]. Mean and median percent improvement thresholds were calculated. Panelist responses to the second administration of the outcome listing (Round 3 for original outcomes; Round 4 for more specific outcomes) and meaningful change exercise (Round 5) were considered as final responses for outcome importance and meaningful change percentages, respectively.

#### Ethics approval and consent to participate.

The “A practical application of the e-Delphi responder definitions” section of the manuscript was a reanalysis of published data from a phase 3, randomized, placebo-controlled trial of leniolisib by V. Koneti Rao, et al (Rao VK, Webster S, Šedivá A, et al. A randomized, placebo-controlled phase 3 trial of the PI3Kδ inhibitor leniolisib for activated PI3Kδ syndrome. Blood. 2023;141:971–983). The original phase 3 trial received approval from independent ethics committees or institutional review boards at each center, and patients and/or their guardians provided written informed consent. Patient data for the reanalysis were accessed April 9, 2024, and all individual patient data were deidentified.

## Results

### Clinician interviews

**Fig 1:**
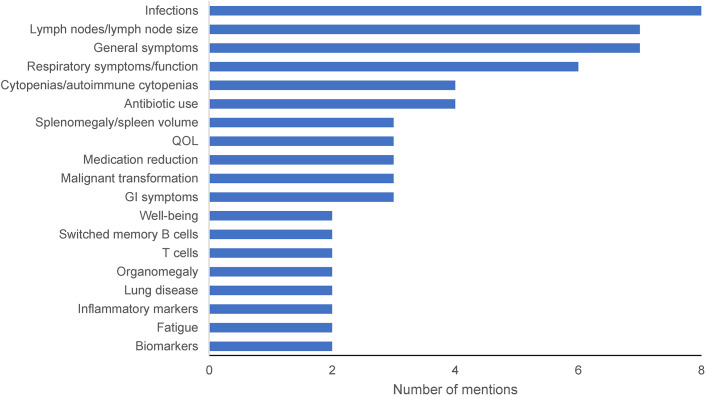
Treatment Outcome Frequencies ≥2 From Clinician Interviews. This figure includes the total number of times that an outcome was mentioned overall. A single outcome may have been mentioned more than once in the interview. Each clinician could provide more than one response; therefore, the sum of the counts in the figure does not equal 9. Outcomes receiving 1 comment included pulmonary function, patient experience, metabolic activity, malignancy, lymphoma risk (with associated comment that a surrogate would be needed to measure), joint pain, inflammation/autoimmune, vaccination response, IgG treatment, hospital visits, fever, exercise intolerance, energy level, EBV, EBV load, easy bleeding and bruising, side effects, coughing, clinical outcomes–general organ involvement, chest therapy/inhaler, bruising, school attendance, autoimmunity symptoms, autoimmune disease, appetite, rash, adenopathy/organomegaly, able to sleep, viral loads, serum immunoglobulins, PD-1^+^CD4^+^ and PD-1^+^CD8^+^, naïve T cells, naïve B cells, memory B cells, CD4/CD8 T-cell ratio, B cells, B-cell function, and all B- and T-cell panel information. Additional single-mention comments included patient heterogeneity, CVID, and unavailability of some outcomes in the clinic. CD, cluster of differentiation; CVID, common variable immunodeficiency; EBV, Epstein-Barr virus; GI, gastrointestinal; Ig, immunoglobulin; PD-1, programmed cell death protein 1; QOL, quality of life.

A range of outcomes were mentioned by the 9 clinicians who participated in the initial interviews. Infections (n = 8), lymph nodes/lymph node size (n = 7), general symptoms (n = 7), and respiratory symptoms/function (n = 6) were the most common individual treatment outcomes (Fig 1). Responses also highlighted the relevance of biomarkers, including cytopenias/autoimmune cytopenias (n = 4), switched memory B cells (n = 2), T cells (n = 2), and inflammatory markers (n = 2). Medication reduction (n = 3) and antibiotic use (n = 4) were common themes, as were quality of life (QOL) (n = 3) and the related concepts of well-being (n = 2) and patient experience (n = 1). Malignancy and organomegaly were also discussed during the interviews.

When the clinicians were asked questions regarding meaningful change on lymph node size (log_10_-transformed SPD of index lesions) and ratio of naïve B cells to total B cells metrics, the most common response was that thresholds of these metrics in APDS were unknown and/or that clinicians were not familiar with these metrics. Among those who noted a specific threshold, 50% reduction was the most commonly mentioned threshold, with other thresholds ranging from 20% to 75% reduction; a return to values within the normal range was also mentioned. Clinicians responded positively to the MDRI that incorporated multiple outcomes into an individual patient profile (n = 5) and to the importance of consistency across outcomes (n = 4), indicating the relevance of observing improvement across multiple treatment outcomes.

For age-related issues that should be considered in the evaluation of treatment outcomes, prevention of future negative outcome(s) (n = 5) was the most common response, which highlighted the importance of early treatment initiation to avoid permanent organ damage secondary to APDS (e.g., pulmonary fibrosis). Other responses related to the theme of avoiding long-term irreversible damage included accumulated tissue injury (n = 2) and fibrosis/scarring (n = 1) in adults and older patients, respectively. When assessing outcomes for specific age groups, growth (n = 2), weight gain (n = 1), school attendance (n = 1), and kids sports (n = 1) were considered important outcomes in children; malignant transformation (n = 1) was noted in adults.

### E-Delphi panel study

**Fig 2:**
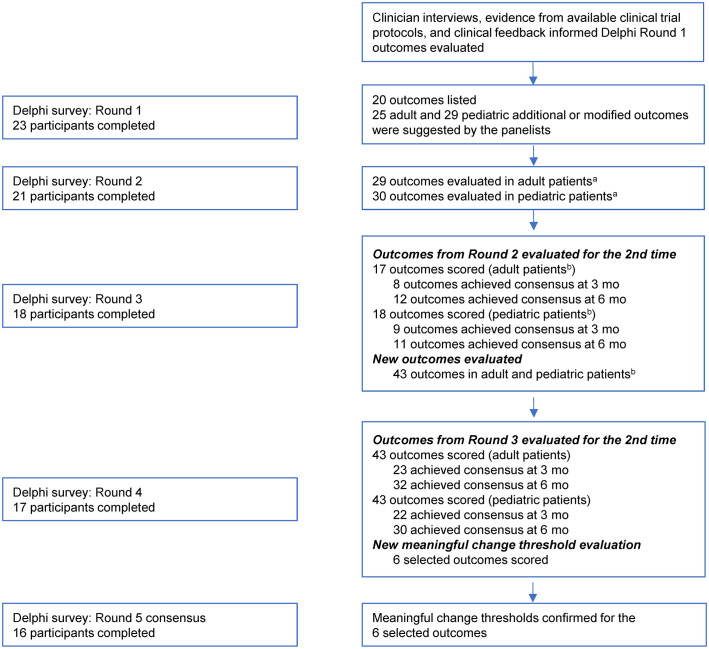
Overview of the E-Delphi Panel Study Process. ^a^Select outcomes from Round 1 were expanded or consolidated to optimize panelist review in Round 2. Outcomes identified as less relevant in adult patients (e.g., growth, height, weight) were removed from consideration but were retained for pediatric patients. ^b^Original outcomes from Round 2 were evaluated for a second time in Round 3; modified items were listed as new and evaluated for the first time.

Fig 2 summarizes the steps taken in the development and conduction of the e-Delphi panel study. Twenty-four clinicians (Table 1) accepted the invitation to participate and 23 (96%) completed the Round 1 survey. Collectively, panelists had treated a mean of approximately 1300 patients with PI and a mean of 5.5 patients with APDS in their careers; notably, the panelists had treated a mean of 2.5 patients in the previous year despite APDS being an ultra-rare disease. Panelists indicated they had high familiarity with the disease, as indicated by the reported mean familiarity score of 8.9 on a scale of 0–10 (0 = no familiarity, 10 = extreme familiarity) (Table 1).

**Table 1:** Panelist Background.

Variable	Panelists (N = 24)
**Location, No. (%)**	
United States	21 (88)
Canada	1 (4)
United Kingdom	1 (4)
Italy	1 (4)
**Specialty area, No. (%)**	
Allergy/Immunology	23 (96)
Hematology/Oncology	1 (4)
**Pediatric specialty, No. (%)**	
Yes	8 (33)
**No. of patients with PI treated in previous year**	
Mean	183.3
Median (range)	135 (0-500)
**No. of patients with PI treated in career**	
Mean	1311.5
Median (range)	800 (0-5000)
**No. of patients with APDS treated in previous year** ^**a**^	
Mean	2.5
Median (range)	2 (0-10)
**No. of patients with APDS treated in career** ^**a**^	
Mean	5.5
Median (range)	3.5 (0-25)
**Familiarity with APDS (0–10 scale; 0 = not familiar at all, 10 = extremely familiar)**	
Mean	8.9
Median (range)	9 (6-10)

APDS, activated phosphoinositide 3-kinase delta syndrome; PI, primary immunodeficiency.

^a^One panelist had not treated a patient with APDS at time of participation in the e-Delphi panel; however, they met the panelist criterion of treating 1 or more patients with a PI. Additionally, their level of familiarity with APDS was an 8 from active involvement in multidisciplinary team management and discussions of patients with APDS.

In Round 1, panelists reviewed a listing of outcomes, proposed new outcomes that could be added to the listing for each subgroup, and provided comments regarding the removal of several outcomes (S1 Table). This input was used to draft the Round 2 survey. The 23 panelists who completed Round 1 were invited to take part in Round 2, with 21 (91%) completing the survey. In Round 2, the majority of outcomes exceeded the ≥ 75% agreement threshold and several were endorsed by 100% of respondents. Organ size/volume and lymph node size/volume were endorsed by all panelists across all the patient subgroups. Conversely, tumor necrosis factor α (TNF-α) as an inflammatory signal was endorsed by <50% of panelists across subgroups. After completion of Round 2, new outcomes with greater specificity were added to Round 3—developed from comments by panel members—to further understand original outcome categories that had been endorsed by ≥75% of panelists. For example, the category “Hematologic parameters: hemoglobin, platelets, lymphocytes, neutrophils” was split into 4 separate categories, 1 for each individual parameter.

**Fig 3:**
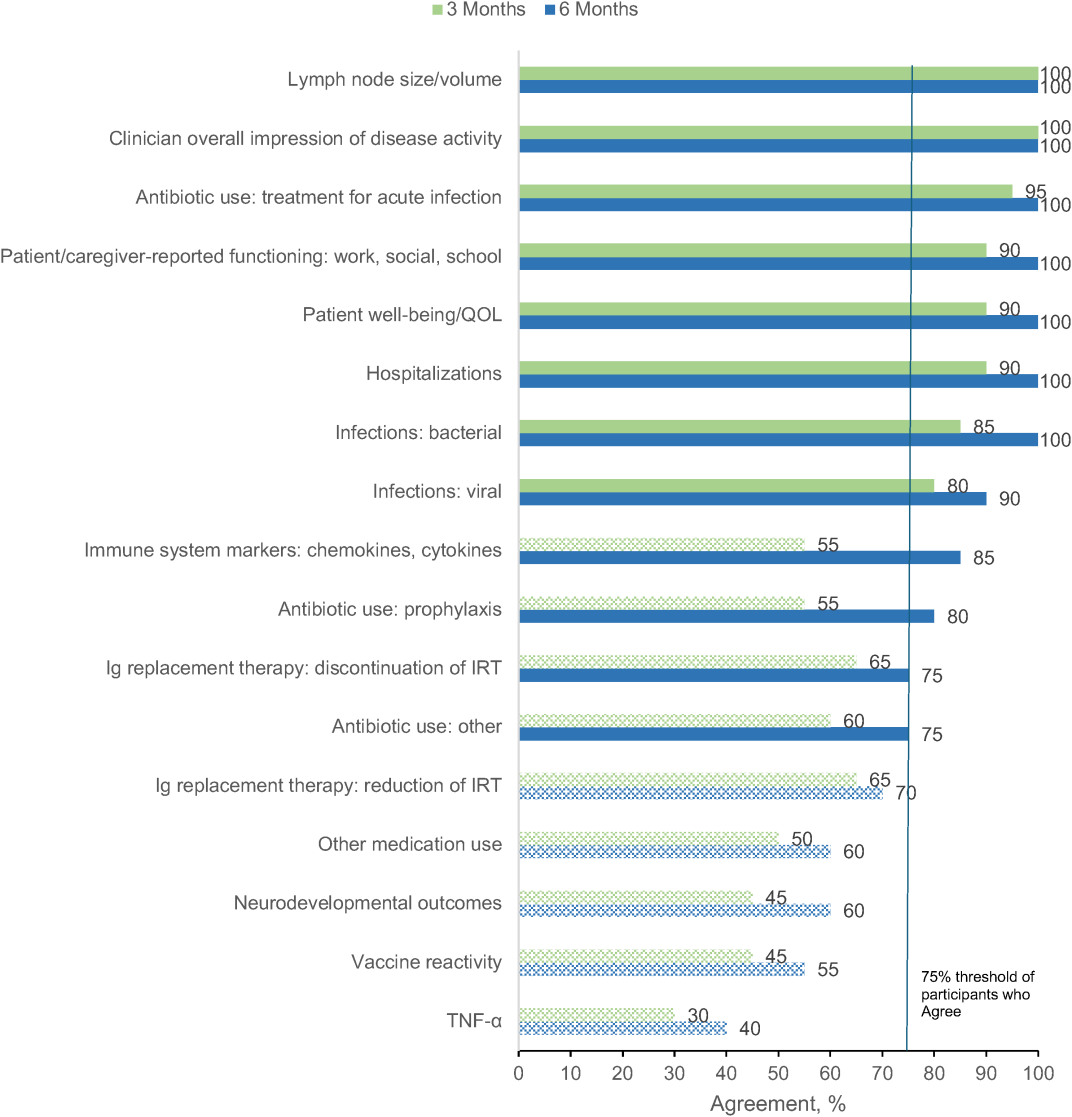
Percent Agreement (Agree or Strongly Agree) for Treatment Outcomes in Adult Patients at 3 Months and 6 Months After Starting Treatment (Round 3). Outcomes not meeting the ≥ 75% threshold for consensus, represented by the vertical blue line, are indicated with pattern fill. Outcomes meeting the ≥ 75% threshold for consensus were considered to be finalized. Ig, immunoglobulin; IRT, immunoglobulin replacement therapy; QOL, quality of life; TNF-α, tumor necrosis factor α.

**Fig 4:**
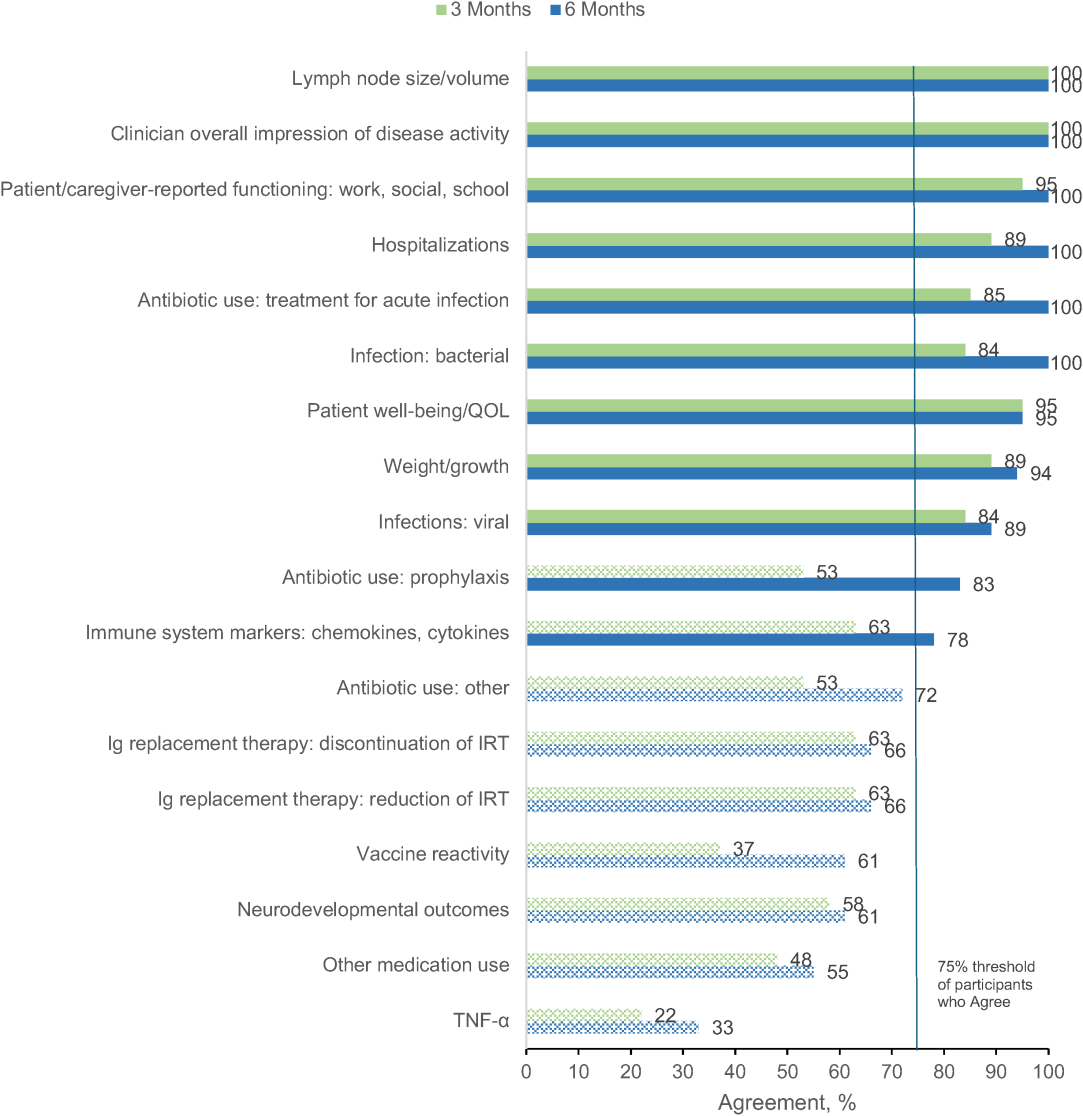
Percent Agreement (Agree or Strongly Agree) for Treatment Outcomes in Pediatric Patients at 3 Months and 6 Months After Starting Treatment (Round 3). Outcomes not meeting the ≥ 75% threshold for consensus, as represented by the vertical blue line, are indicated with pattern fill. Ig, immunoglobulin; IRT, immunoglobulin replacement therapy; QOL, quality of life; TNF-α, tumor necrosis factor α.

The primary goal of Round 3 was to confirm the panelists’ Round 2 agreement ratings regarding whether the original treatment outcomes would be included in their evaluation of a patient starting APDS treatment. The 21 panelists who completed Round 2 were invited to participate in Round 3, with 18 (86%) completing the survey. Figs 3 and 4 show the proportion of panelists who selected “Agree” or “Strongly Agree” for each of the original treatment outcomes in the adult and pediatric populations, respectively. In both pediatric and adult patients, the clinician overall impression of disease activity as well as lymph node size/volume was endorsed by 100% of panelists at both 3 and 6 months after treatment initiation. Patient well-being/QOL, patient/caregiver-reported functioning, hospitalization, infections, and antibiotic use for treatment of acute infection were also represented with high agreement across subgroups. Additionally, weight/growth was identified as an important treatment outcome for pediatric patients with APDS.

**Fig 5:**
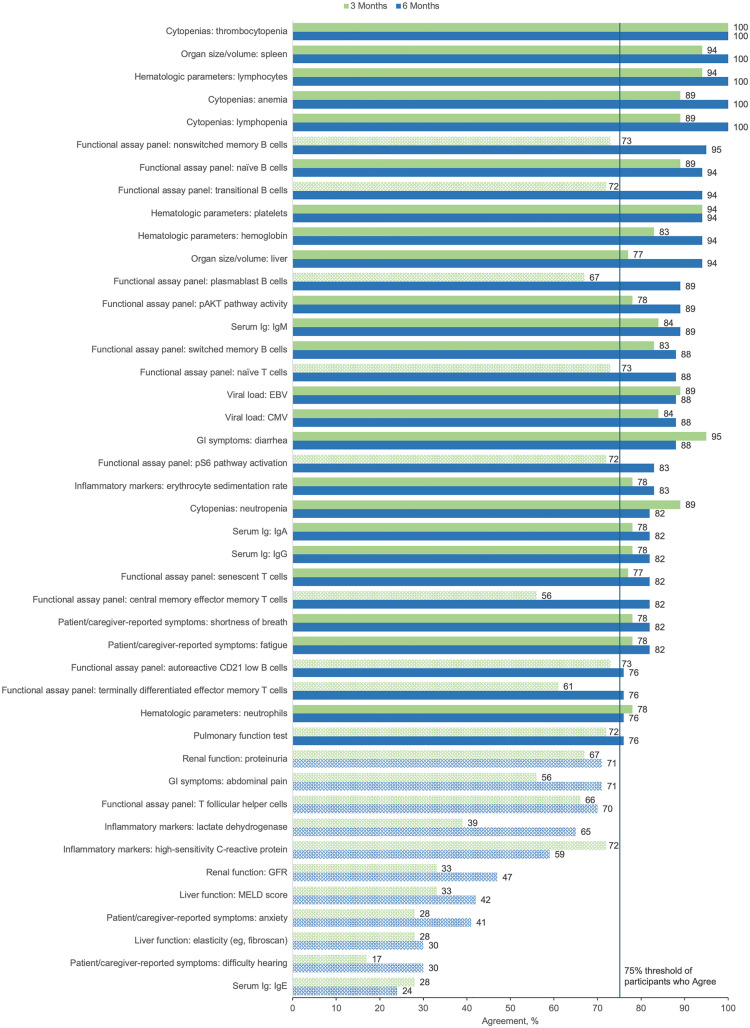
Percent Agreement (Agree or Strongly Agree) for Revised Treatment Outcomes in Adult Patients at 3 Months and 6 Months After Starting Treatment (Round 4). Outcomes not meeting the ≥ 75% threshold for consensus, as represented by the vertical blue line, are indicated with pattern fill. CD, cluster of differentiation; CMV, cytomegalovirus; EBV, Epstein-Barr virus; GFR, glomerular filtration rate; GI, gastrointestinal; Ig, immunoglobulin; MELD, Model for End-Stage Liver Disease; pAKT, phosphorylated protein kinase B; pS6, phospho-S6 ribosomal protein.

**Fig 6:**
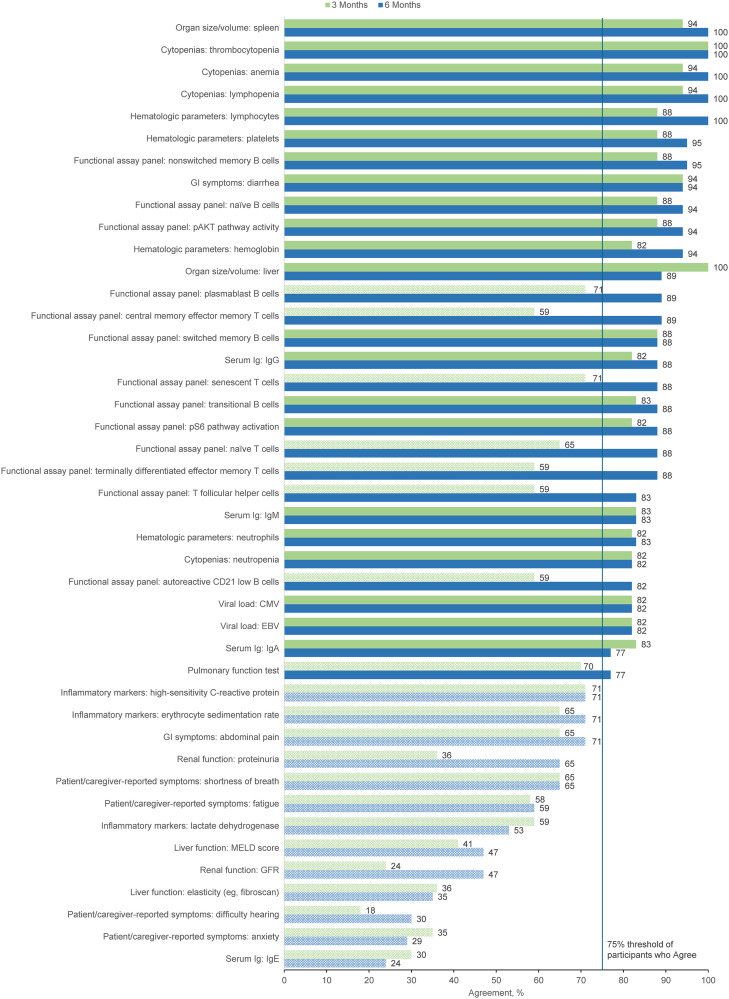
Percent Agreement (Agree or Strongly Agree) for Revised Treatment Outcomes in Pediatric Patients at 3 Months and 6 Months After Starting Treatment (Round 4). Outcomes not meeting the ≥ 75% threshold for consensus, represented by the vertical blue line, are indicated with pattern fill. CD, cluster of differentiation; CMV, cytomegalovirus; EBV, Epstein-Barr virus; GFR, glomerular filtration rate; GI, gastrointestinal; Ig, immunoglobulin; MELD, Model for End-Stage Liver Disease; pAKT, phosphorylated protein kinase B; pS6, phospho-S6 ribosomal protein.

The primary goal of Round 4 was to confirm level of agreement with the panelists’ responses to new treatment outcomes presented in the Round 3 survey. Nineteen panelists were invited to participate in Round 4, which included the 18 panelists who completed the Round 3 survey and 1 panelist who was unable to complete the Round 3 survey but asked to be invited to Round 4. Of the 19 panelists, 17 (89%) completed the Round 4 survey. Figs 5 and 6 show the proportion of panelists who selected “Agree” or “Strongly Agree” for each of the new specific treatment outcomes in the adult and pediatric populations, respectively. Respondents endorsed >50% of treatment outcomes using an agreement threshold of ≥75% across outcomes categories and age groups. This percentage increased to ≥70% 6 months after starting treatment in adult and pediatric patients.

The Round 5 survey was designed to evaluate meaningful improvements in 6 selected treatment outcomes identified in Round 4: lymph nodes, naïve B cells, spleen volume, hemoglobin, platelets, and lymphocytes. The 19 panelists who were invited to complete Round 4 were also invited to participate in Round 5, with 16 (84%) completing the survey. Median values for meaningful changes in the 6 selected outcomes were between 20% and 45% (Table 2). Panelists indicated within-patient clinically meaningful improvements in adult patients ranged from median values of 20% to 25% at 3 months and 25% to 30% at 6 months in the 6 selected treatment outcomes. Similarly, panelists indicated within-patient clinically meaningful improvements in pediatric patients ranged from median values of 20% to 27.5% at 3 months and 22.5% to 45% at 6 months. In both adult and pediatric patients, panelists indicated that higher thresholds were required at 6 months than at 3 months for within-patient improvements to be considered clinically meaningful. Thresholds were generally similar across outcomes and between age groups, except for lymph node size reduction. At 6 months, the median reduction in lymph node size in pediatric patients was 45%, which was 15% higher than reductions observed in adult patients, whereas no other outcome threshold exceeded 35%.

**Table 2:** Within-Patient Meaningful Change Percentages and Panelist Endorsement of a Benchmark for Each Selected Outcome (Round 5) (n = 16).

	Lymph nodes	Naïve B cells	Spleen volume	Hemoglobin	Platelets	Lymphocytes
**Adult, 3 mo**						
Percent improvement						
Mean (SD)	26.6 (12.6)	21.3 (8.7)	27.5 (12.9)	19.7 (10.2)	21.6 (8.9)	20.3 (6.2)
Median (IQR)	20.0 (10.0)	20.0 (0.0)	25.0 (10.0)	20.0 (11.3)	20.0 (5.0)	20.0 (5.0)
Percentage of panelists endorsing a benchmark	6	6	13	25	19	13
**Adult, 6 mo**						
Percent improvement						
Mean (SD)	34.7 (14.7)	28.8 (11.6)	34.1 (14.1)	28.8 (13.2)	30.6 (13.2)	28.1 (11.7)
Median (IQR)	30.0 (26.2)	25.0 (2.5)	27.5 (25.0)	25.0 (11.2)	25.0 (22.5)	25.0 (2.5)
Percentage of panelists endorsing a benchmark	13	19	19	25	38	13
**Pediatric, 3 mo**						
Percent improvement						
Mean (SD)	24.7 (11.3)	23.4 (8.7)	25.3 (9.7)	19.7 (10.2)	22.8 (9.3)	22.2 (9.7)
Median (IQR)	25.0 (5.0)	20.0 (5.0)	27.5 (10.0)	20.0 (11.3)	20.0 (6.3)	22.5 (6.3)
Percentage of panelists endorsing a benchmark	6	13	19	25	25	13
**Pediatric, 6 mo**						
Percent improvement						
Mean (SD)	38.4 (13.8)	30.9 (14.2)	35.6 (14.4)	30.0 (16.2)	31.9 (15.5)	27.5 (12.0)
Median (IQR)	45.0 (25.0)	25.0 (30.0)	35.0 (25.0)	22.5 (15.0)	30.0 (15.0)	25.0 (6.3)
Percentage of panelists endorsing a benchmark	6	19	25	38	31	13

IQR, interquartile range; SD, standard deviation.

Panelists were then asked if they thought a benchmark existed for each outcome (yes/no). Panelists (%, across subgroups) most often indicated a benchmark for hemoglobin level (25%−38%) and platelet count (19%−38%) (Table 2). The most common response by panelists proposing a benchmark was that values should be “normal” or within the “normal range.”

### A practical application of the e-Delphi responder definitions

The median within-patient meaningful change percentages endorsed by the panelists (Table 2) were retrospectively applied to the results of a randomized controlled phase 3 trial (NCT02435173) that compared leniolisib with placebo in the treatment of patients with APDS [20] to determine the proportion of adult (aged ≥18 years) and adolescent (aged 12 to <18 years) patients with APDS who experienced clinically meaningful within-patient changes at 3 months. Six treatment outcomes were analyzed for adult and pediatric patients and included percentage decrease (adult, 20%; pediatric, 25%) in lymph node size in patients with enlarged lymph nodes at baseline; percentage decrease (adult, 25%; pediatric 27.5%) in spleen volume in patients with enlarged spleen volume (>314,000 mm^3^) at baseline [25]; percentage increase (adult, 20%; pediatric 20%) in naïve B-cell to total B-cell ratio; and the percentage increase of lymphocyte (adult, 20%; pediatric, 22.5%) and platelet (adult, 20%; pediatric, 20%) counts and hemoglobin level (adult, 20%; pediatric, 20%) in patients with baseline measurements that were not within reference ranges. Change from baseline was measured from the baseline visit to the day 85 visit. If day 85 data were not available, the closest visit before day 85 was used. This study used SAS software to perform a statistical comparison between leniolisib and placebo, represented by the Fisher exact test.

**Fig 7:**
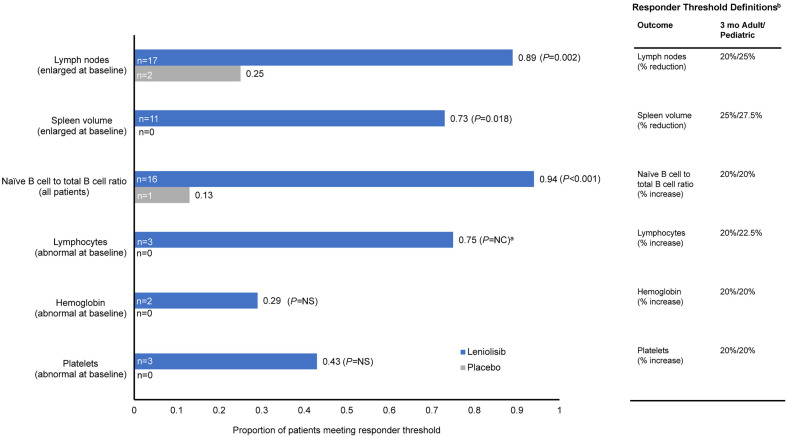
Meeting Responder Thresholds at 3 Months in 6 Outcomes Using Data From a Randomized Phase 3 Study [20]. ^a^**A statistical comparison between the proportion of responders in the leniolisib and placebo treatment arms was not conducted for lymphocytes, as there were no patients in the placebo arm with an abnormal lymphocyte count at baseline. **^b^**Responder threshold definitions were selected through an e-Delphi panel of APDS clinicians.** APDS, activated phosphoinositide 3-kinase delta syndrome; NC, not calculable; NS, not significant.

Baseline patient characteristics from the randomized phase 3 trial are provided in Table 3. Comparisons of patients meeting e-Delphi–derived responder thresholds at 3 months are provided in Fig 7. Significant between-group differences, favoring leniolisib over placebo, were observed including improvements in lymph node size in patients with enlarged lymph nodes at baseline (*P* = 0.002), spleen volume in patients with an enlarged spleen at baseline (*P* = 0.018), and naïve B-cells to total B-cells ratio in all patients (*P* < 0.001). Numerical improvements favoring leniolisib over placebo were observed for hemoglobin levels and platelet counts in patients with abnormal baseline values; however, differences between groups were not statistically significant. Because no patients in the placebo arm had abnormal lymphocyte counts at baseline, between-group comparisons were not performed.

**Table 3:** Patient Characteristics From a Randomized Phase 3 Trial [20].

Characteristic	Leniolisib (n = 21)	Placebo (n = 10)
**Age**		
Median (range), y	20.0 (12-54)	19.5 (15-48)
<18 y, n (%)	8 (38.1)	4 (40.0)
**Male/female sex, %**	52.4/47.6	40.0/60.0
**Weight, median (range), kg**	67.1 (46.9-100.6)	68.9 (50.0-88.0)
**No. of patients with lymph node size enlarged at baseline**	19	8
**No. of patients with spleen volume enlarged at baseline**	15	4
**No. of patients with ratio of naïve B cells to total B cells**	17	8
**No. of patients with lymphocyte count outside of reference range at baseline**	4	0
**No. of patients with hemoglobin level outside of reference range at baseline**	7	3
**No. of patients with platelet count outside of reference range at baseline** ^**a**^	7	1

^a^All patients presented with thrombocytopenia at baseline except 2 patients with elevated platelet counts (above the upper level of normal) at baseline.

## Discussion

Delphi panel studies have been used in health care research as a rigorous way to obtain consensus opinion of a group of experts without obstacles such as dominance and group conformity [22]. These studies can be beneficial in rare and ultra-rare diseases such as APDS, in which treatment outcomes and thresholds for meaningful treatment responses have not been clearly defined [26,27]. In this study, we engaged experts, through one-on-one interviews and an e-Delphi panel study, to reach a consensus regarding the most important outcomes to consider and the amount of change in a treatment outcome that would indicate a meaningful benefit for an individual patient (i.e., a responder definition).

A preliminary assessment of meaningful outcomes in APDS was obtained from the initial clinician interviews, and results suggested that several outcomes in APDS were considered clinically important. However, interview responses indicated a lack of knowledge of thresholds for metrics such as lymph node size (log_10_-transformed SPD of index lesions) and ratio of naïve B cells to total B cells was observed. To formally evaluate treatment outcomes and determine thresholds for meaningful change, an e-Delphi panel study was designed and conducted with APDS experts.

The e-Delphi panel study included 23 panelists completing ≥1 round. Retention of participants was generally high, with 16 of 23 (70%) completing the Round 5 survey; panelists were only excluded from the Delphi process for failure to complete a survey. All panelists had experience treating ≥1 patient with APDS or PI in their career and had a very high level of familiarity with APDS. The e-Delphi panel endorsed numerous patient-related and clinical outcomes with ≥75% agreement, including biomarkers, clinical measures, and patient- or caregiver-reported measures of symptoms and QOL. Specifically, 100% of panelists agreed on outcomes of lymph node size/volume and clinician overall impression of disease activity at 3 and 6 months after treatment initiation in both adult and pediatric patients. Other outcomes that met consensus—for adult and pediatric patients after treatment initiation—included patient/caregiver-reported functioning at work, social, and school; antibiotic use for acute infections; patient well-being/QOL; hospitalizations; and viral and bacterial infections. Weight/growth in pediatric patients at 3 and 6 months after treatment initiation also reached consensus. Hematologic parameters and associated clinical manifestations (e.g., cytopenias) were also widely endorsed as were spleen and liver volume. Outcomes endorsed by <75% of panelists, including TNF-α, vaccine reactivity, and patient- or caregiver-reported hearing difficulty and anxiety, were not considered important outcomes. Additionally, reduction and discontinuation of immunoglobulin replacement therapy (IRT) did not meet the 75% threshold for consensus. This might be due to the short time interval of 3 or 6 months, which some panelists indicated was insufficient time to implement changes to an IRT regimen or to assess vaccine reactivity. Panelists also noted that not all patients receive IRT before starting a treatment.

Across all subgroups in the e-Delphi panel study (pediatric and adult, at 3 and 6 months), 100% of panelists endorsed lymph node size as an important treatment outcome, and 88% to 94% of panelists endorsed naïve B-cell count when evaluating a treatment. These results support the use of these outcomes as end points in trials evaluating leniolisib, the only treatment approved by the US Food and Drug Administration for APDS [28]. For example, in the phase 3 trial of leniolisib, lymph node size, percentage of naïve B cells, spleen volume, and key immune cell subsets were improved to a significantly greater degree in patients assigned to leniolisib than in patients who received placebo [20].

E-Delphi panelists also indicated the amount of improvement on 6 selected outcomes (lymph node size, naïve B-cell count, spleen volume, hemoglobin levels, platelet count, and lymphocyte count) that they would consider to be clinically meaningful. The median thresholds were between 20% and 30% for almost all outcomes and across subgroups. Only lymph node size and spleen volume at 6 months for pediatric patients were higher at 45% and 35%, respectively. Overall, threshold values were slightly higher at 6 months than at 3 months, reflecting the expectation that values should continue to improve with time. A minority of panelists indicated that there was an absolute value benchmark that they would use instead of a percent improvement threshold. This was most commonly reported for hemoglobin levels, in which returning to “normal” levels might be considered an important treatment goal. Alternative reference targets mentioned by panelists, but not meeting consensus, included those from diseases such as immune thrombocytopenia and hemolytic anemia. Lymphadenopathy, which is a leading sign of APDS and is present in almost all patients with APDS, often co-occurs with splenomegaly [29]. The pervasiveness of lymphadenopathy in APDS enables its use as a surrogate marker of immune dysregulation [29].

Given the ultra-rare nature of APDS, clinicians may never see a patient with APDS during their career. Our expert e-Delphi panelists had collectively treated a mean of 2.5 patients with APDS within the past year, a mean of 5.5 patients over their career, and reported having a high level of familiarity with APDS, making them well suited to participate in this study. Although the majority of panelists were allergists and immunologists, only 1 panelist was a hematology/oncology specialist, which could limit the generalizability of the results outside of the immunology setting. However, most patients with APDS or PI/IEI are treated in allergy/immunology clinics, suggesting the makeup of the panel reflects real-world experience. During the study, panelists had access to published leniolisib trial results. However, this study was designed to broadly assess APDS treatments and included no specific drug reference. Although panelists could review aggregated comments in Rounds 3 and 4, an integral part of the Delphi method, there was minimal opportunity for interaction and elicitation of feedback regarding why they assigned importance to certain outcomes and why they selected specific meaningful change thresholds [23]. This limited interaction mitigated any undue influence of panelists on each other, especially by more senior physician panelists. Furthermore, the large number of potential outcomes made it infeasible to obtain meaningful change estimates for all variables. As our study limited treatment outcomes at 3 and 6 months after starting therapy, the effect of treatment on the lifetime risks of developing lymphoma or bronchiectasis progressing to end-stage lung disease were not considered, despite the important connection with serious morbidity and premature death in patients with APDS. Additionally, the short time frame assessed did not allow for meaningful assessment of IRT reduction or discontinuation. Future research of other potential outcomes, including evaluation of specific APDS treatments, and research using longer time frames for treatment assessment are warranted.

When responder thresholds were retrospectively applied to results from a phase 3 trial that compared leniolisib with placebo in patients with APDS, treatment with leniolisib resulted in significant and meaningful improvements in disease hallmarks including lymph node size, spleen volume, and the ratio of naïve B cells to total B cells at 3 months [20]. Despite the low numbers of patients experiencing certain cytopenias (anemia, thrombocytopenia, or lymphopenia) at baseline, some improvements were observed at 3 months.

Thus, the most important outcomes identified by the e-Delphi panelists can be considered for use in the clinic to guide any APDS treatment. The results of this study may also help inform clinical practice by identifying biomarkers and clinical outcomes, as well as meaningful within-patient changes, to advise decision-making in the clinic.

## Supporting information

S1 Table Treatment outcomes in adult patients at 3 and 6 months after start of treatment, e-Delphi panel study round 1. (DOCX)

## Abbreviations

APDS: activated phosphoinositide 3-kinase delta syndrome;
CD: cluster of differentiation
CMV: cytomegalovirus
CVID: common variable immunodeficiency
EBV: Epstein-Barr virus
ESR: erythrocyte sedimentation rate
GFR: glomerular filtration rate
GI: gastrointestinal
IEI: inborn error of immunity
Ig: immunoglobulin
IRT: immunoglobulin replacement therapy
MELD: Model for End-Stage Liver Disease
MDRI: multidomain response index
pAKT: phosphorylated protein kinase B
PD-1: programmed cell death protein 1
PI: primary immunodeficiency
PI3Kδ: phosphoinositide 3-kinase delta
*PIK3CD*: phosphatidylinositol-4,5-bisphosphate 3-kinase catalytic subunit delta
*PIK3R1*: phosphoinositide-3-kinase regulatory subunit 1
pS6: phospho-S6 ribosomal protein
QOL: quality of life
SPD: sum of the perpendicular diameters
TNF-α: tumor necrosis factor α
