# Supplementary material for: Key outcomes in treatment of activated phosphoinositide 3-kinase delta syndrome: An e-Delphi panel study and responder threshold application
Source: PLoS One. 2025 Oct 15;20(10):e0333341. doi: 10.1371/journal.pone.0333341 (PMC12527126; doi:10.1371/journal.pone.0333341)
Supplement: S1 Table — (DOCX) [file pone.0333341.s001.docx]

**Supporting Information**

**S1 Table. Treatment Outcomes in Adult Patients at 3 and 6 Months After Start of Treatment, E-Delphi Panel Study Round 1**

| **Outcomes for adult and pediatric patients at 3 and 6 months after starting treatment—provided to panelists in Round 1** |
| --- |
| Antibiotic use |
| B cells: percentage of naïve B cells out of total B cells |
| Other B cells: CD38^+^ plasmablasts, mature, nonswitched memory, switched memory, transitional |
| Clinician overall impression of disease activity |
| Cytopenias: anemia, thrombocytopenia, lymphopenia, neutropenia |
| Hematologic parameters: hemoglobin, platelets, lymphocytes, neutrophils |
| Immune system markers: chemokines, cytokines |
| IRT use |
| Infections |
| Inflammatory markers: erythrocyte sedimentation rate, high-sensitivity C-reactive protein, lactate dehydrogenase |
| Lymph node size/volume |
| Other medication use |
| Organ size: spleen, liver |
| Patient well-being/quality of life |
| Patient/caregiver-reported symptoms |
| Patient/caregiver-reported functioning: work, social, school |
| Serum immunoglobulins: IgA, IgG, IgM |
| T cells: CD3^+^, CD4^+^, CD8^+^, naïve, central memory effector memory, terminally differentiated effector memory, senescent, PD-1^+^ |
| TNF-α |
| Viral load: CMV load, EBV load |
| **Additional or modified outcomes in adult patients at 3 and 6 months after starting treatment—proposed by the panelists during Round 1** |
| Pulmonary function |
| Chest CT |
| Presence of parenchymal lung disease and/or bronchiectasis |
| Specific antibody response to vaccination |
| Response to protein and polysaccharide vaccines |
| Vaccine titers: pneumococcal titers |
| Specific antibody function |
| In vitro T-cell proliferation |
| Soluble CD25 level |
| CD8^+^CD57^+^ T-cell enumeration |
| pS6 in T cells |
| T follicular helper cells |
| GI outcomes: remission of inflammatory bowel disease, weight gain, etc |
| GI symptoms |
| GI symptoms: diarrhea/vomiting |
| Risk of developing lymphoma |
| Presence of organ-specific lymphoid aggregates, especially mucosal |
| Need for hospitalization for infection |
| Weight change |
| Growth parameters |
| Endocrine parameters |
| Development of lymphoma |
| Medication side effects |
| Need for ED or urgent care visit for infection, fever |
| Neuropsych symptoms |
| **Additional or modified outcomes in pediatric patients at 3 and 6 months after starting treatment—proposed by the panelists during Round 1** |
| Lung function testing |
| Pulmonary function testing |
| Pulmonary function |
| Chest CT |
| Specific antibody response to vaccines |
| Vaccine titers |
| Vaccine titers: pneumococcal titers |
| Specific antibody function |
| In vitro T-cell proliferation |
| Soluble CD25 level |
| Enumeration of CD8^+^CD57^+^ T cells |
| Gastrointestinal symptoms |
| GI symptoms |
| Diarrhea |
| Risk of developing lymphoma |
| Hospitalizations |
| Need for hospitalization for infection, fever |
| Need for ED or urgent care visit for infection, fever |
| Weight change |
| Growth |
| Growth, specifically weight |
| Growth parameters |
| Growth parameters (height/weight) |
| Growth chart |
| Endocrine parameters |
| Side effects of drug |
| Neurodevelopmental assessments |
| T follicular helper cells |
| Neuropsych symptoms |

CD, cluster of differentiation; CMV, cytomegalovirus; CT, computed tomography; EBV, Epstein-Barr virus; ED, emergency department; Ig, immunoglobulin; GI, gastrointestinal; IRT, immunoglobulin replacement therapy; PD-1, programmed cell death protein 1; pS6, phopho-S6 ribosomal protein; TFN-α, tumor necrosis factor alpha.
